# Nanobots: An endodontist saviour

**DOI:** 10.6026/973206300200898

**Published:** 2024-08-31

**Authors:** Saurav Bathla, Manoj Kumar Hans, Saurav Kumar Dutta, Shyamalima Bhattacharyya, Anindita Talukdar, Sana Saifi

**Affiliations:** 1 Department of Conservative Dentistry and Endodontics, Institute of Dental Sciences, Bareilly, Uttar Pradesh, India; 2 Department of Public Health Dentistry, Regional Dental College, SSUHS, Guwahati, India; 3 Department of Pediatric & Preventive Dentistry, Regional Dental College, SSUHS, Guwahati, India

**Keywords:** Dentinal tubules, *Enterococcus faecalis*, Magnetic field

## Abstract

The application of nanoparticles in the form of solution for irrigation, medication and as an additive for sealer/restorative material has been evaluated to improve the antibacterial efficacy in the field of endodontics. Recently developed nanobots are injected into the teeth to destroy pathogens and they are more effective in root canal therapy. They are helical shaped and composed of silicon dioxide with iron embedded into the silica body to provide magnetic properties. They are also able to move within the dentinal tubules and are manipulated peripherally through a low-intensity magnetic field. These nanobots can be administered into the cleaned central canal of a tooth samples which are immersed in deionized water and their motions are monitored using a near-infrared imaging technique to produce heat which is a hyperthermia-based bactericidal method which help to eradicate the surrounding pathogens.

## Description:

Dental caries, trauma and tooth malformations frequently cause an infection of the dental pulp which results in pulpitis, pulp necrosis and finally apical periodontitis if left untreated. In that case, root canal treatment (RCT) is indicated, as the removal of necrotic pulp tissue and bacteria from the entire root canal system enables healing of periapical pathosis [1, 2-3]. However, biomechanical preparation independent of the technique or type of irrigation used provides a significant reduction of the microbiota but only in the main root canal [4]. Even after completion of biomechanical preparation, the microorganisms remain persisted in dentinal tubules, isthmuses, lateral canals and accessory canals which may reinfect the root canal [5]. The extent of penetration of microorganisms into the dentinal tubules of infected root canals reported in the literature varied from 200-1,500µm (Table 1) [6, 7, 8, 9, 10, 11, 12, 13, 14, 15, 16-17]. In fact, within oval canals, only 40% of the root canal wall can be contacted by instruments when a rotating technique is used. Therefore, irrigation is an essential part of a root canal treatment as it allows for cleaning beyond the root canal instruments [18]. The primary objective of chemo-mechanical preparation of root canal systems is elimination of pulpal tissue, inorganic and organic debris, bacteria and their toxic by-products through the use of instruments and irrigation devices [19].

The success of endodontic therapy is based on the combination of adequate instrumentation, irrigation and three dimensional obturation of the root canal system. Approximately 2.3 billion individuals are affected by dental caries globally and about 24 to 50 million procedures are performed annually in the United States alone, of which more than 10% are reported to fail due to the persistence of microbes deep into the dentinal tubules. It is really challenging to remove bacteria from dentinal tubules using traditional methods, because the dentinal tissue has a complicated and constrained morphology [20]. Ultrasonic or laser pulses were employed to produce shockwaves in the fluid used to flush away the pathogens and tissue debris to increase the effectiveness of root canal therapy. But the energy of these pulses quickly dissipated and they can only travel approximately up to 900 micro-meters (Table 2) [21, 22, 23, 24, 25- 26]. Currently available nanobots can be injected into the teeth to destroy pathogen and they are more effective in root canal therapy. They are helical shaped and composed of silicon dioxide with iron embedded into the silica body to provide magnetic properties [27]. Moreover, the amount of silica used in the nanobots is negligible that it can be considered harmless for the human body. They are able to move within the dentinal tubules, manipulated extraneous through the low-intensity magnetic field. They are immersed in water or in a biocompatible medium that resembles water. A billion of them can fit in 0.5 ml of water drop. These nanobots can be administered into the cleaned central canal of a tooth samples which are immersed in deionized water, and their motions are monitored using a near-infrared imaging approach. The nanobots can reach up to 2,000 micro-meters and produce heat which is a hyperthermia-based bactericidal method which might eradicate the surrounding pathogens (Table 2) [27]. They were exposed to rotating magnetic fields, to retrieve them and reduce the chances of nanoparticle accumulation and toxicity. Thus, a hyperthermia-based bactericidal method provides a safer alternative to chemicals or antibiotics. Enterococcus faecali is the most pronounced resistant bacteria isolated from the infected root canal. A nanobot with a rotating magnetic field into an infected tooth sample produced heat and within 15 minutes of exposure pathogen were destroyed [27].

## Conclusion:

Available developed dental nanobots which is a hyperthermia-based bactericidal method provides a safer alternative to chemicals or antibiotics in root canal therapy. Further, the dental nanobots had been tested in animal models and proven to be safe and efficient.

## Future clinical implications:

In the future, the nanobots can aid in surgical procedures, pulpal regeneration, inducing anesthesia, curing tooth hypersensitivity, non-vital tooth bleaching and targeted drug delivery.

## Figures and Tables

**Table 1:** Depth of penetration of microorganisms into the dentinal tubules of infected root canals

**S.No**	**Author(s)**	**Maximum depth of penetration**	**References**
1)	Kouchi Y *et al.* (1980)	1050-1150 µm	[6]
2)	Haapasalo M & Orstavik D. (1987)	800-1000 µm	[7]
3)	Safavi KE *et al.*(1989)	50-300 µm	[8]
4)	Orstavik D & Haapasalo M. (1990)	600-1200 µm	[9]
5)	Horiba N *et al.*(1990)	300-800 µm	[10]
6)	Perez F *et al.*(1993)	Up to 800 µm	[11]
7)	Peters LB *et al.*(1995)	Up to 375 µm	[12]
8)	Berutti E *et al.*(1997)	Up to 300 µm	[13]
9)	Al-Nazhan S *et al.*(2014)	200-1,500 µm	[14]
10)	Vatkar NA *et al.*(2016)	>1000 µm	[15]
11)	Rosen E *et al.*(2020)	Up to 1480 µm	[16]
12)	Kumar PS *et al.*(2021)	800 to 1062 µm	[17]

**Table 2:** Penetration depth of root canal irrigants into dentinal tubules

**S. No**	**Type of irrigants activation methods**	**Maximum depth of penetration**	**References**
1)	Conventional needle irrigation	31-280 µm	[21]
2)	Sonic irrigation	110-337 µm	[21]
3)	Ultrasound irrigation	160-330 µm	[21, 22]
4)	Laser-PIPS (photon induced photoacoustic streaming)	Up to 900 µm	[23, 24-25]
5)	Laser-SWEEPS (shock-wave enhanced emission photoacoustic streaming)	650 - 800 µm	[26]
6)	Magnetic nanobots	Up to 2000 µm	[27]
